# For flux's sake: General considerations for energy‐flux calculations in ecological communities

**DOI:** 10.1002/ece3.8060

**Published:** 2021-09-14

**Authors:** Malte Jochum, Andrew D. Barnes, Ulrich Brose, Benoit Gauzens, Marie Sünnemann, Angelos Amyntas, Nico Eisenhauer

**Affiliations:** ^1^ German Centre for Integrative Biodiversity Research (iDiv) Halle‐Jena‐Leipzig Leipzig Germany; ^2^ Institute of Biology Leipzig University Leipzig Germany; ^3^ School of Science University of Waikato Hamilton New Zealand; ^4^ Institute of Biodiversity University of Jena Jena Germany

**Keywords:** biodiversity and ecosystem functioning, community ecology, energy flow, food web, multitrophic ecosystem functioning, networks

## Abstract

Global change alters ecological communities with consequences for ecosystem processes. Such processes and functions are a central aspect of ecological research and vital to understanding and mitigating the consequences of global change, but also those of other drivers of change in organism communities. In this context, the concept of energy flux through trophic networks integrates food‐web theory and biodiversity‐ecosystem functioning theory and connects biodiversity to multitrophic ecosystem functioning. As such, the energy‐flux approach is a strikingly effective tool to answer central questions in ecology and global‐change research. This might seem straight forward, given that the theoretical background and software to efficiently calculate energy flux are readily available. However, the implementation of such calculations is not always straight forward, especially for those who are new to the topic and not familiar with concepts central to this line of research, such as food‐web theory or metabolic theory. To facilitate wider use of energy flux in ecological research, we thus provide a guide to adopting energy‐flux calculations for people new to the method, struggling with its implementation, or simply looking for background reading, important resources, and standard solutions to the problems everyone faces when starting to quantify energy fluxes for their community data. First, we introduce energy flux and its use in community and ecosystem ecology. Then, we provide a comprehensive explanation of the single steps towards calculating energy flux for community data. Finally, we discuss remaining challenges and exciting research frontiers for future energy‐flux research.

## Graphical abstract

While the concept of energy flux through trophic networks has been around for decades and continued advances in the field have made the approach increasingly accessible, the adoption of this approach and methodology is not necessarily straight forward, especially for people new to the topic. Here, we provide a comprehensive, step‐wise introduction towards calculating energy flux for community data. We discuss solutions for common challenges and offer exciting frontiers for future energy‐flux research.

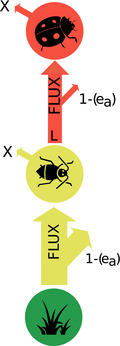

## 1 INTRODUCTION

Global biodiversity is changing with consequences for the functioning of ecosystems and the services they provide to humanity (Cardinale et al., 2012; IPBES, 2019). To assess the potential consequences of this biodiversity change, much research has focused on the relationship between the biodiversity and functioning of ecosystems (Balvanera et al., 2006; Cardinale et al., 2012; Hooper et al., 2005; Loreau et al., 2001). Strikingly, interaction networks of organisms play a key role in connecting community structure and diversity to ecosystem functioning (Brose et al., 2016; Eisenhauer et al., 2019). In this respect, trophic networks (i.e., food webs) have been used to connect structure and function in ecology (Odum, 1968; Odum et al., 1962; Thompson et al., 2012) by connecting multidiversity to multifunctionality through trophic interactions (Barnes et al., 2018). The interaction structure (network topology) alone—who eats whom—can tell us a lot about the properties of the community (e.g., its stability, MacArthur, 1955; Neutel et al., 2002). However, the consideration of quantitative networks, describing the strengths of interactions between the trophic nodes linked to each other in such food webs, enables us to infer rates of ecological processes (Barnes et al., 2018; Odum, 1968; Potapov et al., 2019a) thus providing answers not only on the "who" interacts with each other but also to the "how much" do they interact. This additional layer of information enables us to combine structure and function, connecting diversity and composition to ecosystem processes and services, in order to more comprehensively answer big questions in ecological research.

One exciting way of connecting multitrophic biodiversity to multifunctionality by using such quantitative networks is calculating energy fluxes through interaction networks (Barnes et al., 2018; de Ruiter et al., 1993; Hunt et al., 1987; Lindeman, 1942; Odum, 1968; O'Neill, 1969). By combining food‐web theory with metabolic theory (Barnes et al., 2018), this approach allows the quantification of the energetic backbone for a given community. Specifically, the method allows the estimation of energy transfer from one node (e.g., species) in the food web to another. The information drawn from such energy‐flux calculations can be used as efficient proxies for many different ecosystem processes (Odum, 1968) with several being directly related to functions and services (Barnes et al., 2018; Potapov et al., 2019a; Schwarz et al., 2017). For example, the combined flux of energy to all predators, herbivores, or decomposers in a community can serve as a proxy for predation, herbivory, or decomposition, respectively (Barnes et al., 2018; Potapov et al., 2019a). As such, energy flux represents an inherently multitrophic concept of assessing ecosystem processes that enables us to study the relationship between multitrophic biodiversity and ecosystem functioning (BEF, Barnes et al., 2014) or the altered performance of ecosystems under different climate regimes (Sohlström et al. in press), and perturbations (Schwarz et al., 2017). It also allows the assessment of top‐down versus bottom‐up forcing in a system (Barnes et al., 2020). Moreover, single energy‐flux related processes can jointly be used to calculate trophic multifunctionality (Potapov et al., 2019a) or be combined with other, nontrophic, processes to gain a more general picture of ecosystem multifunctionality (Barnes et al., 2018). Combining such information on trophic and nontrophic processes with scenarios of ecosystem function or service weightings preferred in a given context (e.g., by relevant stakeholders), will be a powerful tool for effective management of multifunctional landscapes (Allan et al., 2015) or to make informed decisions in regard to conservation.

More generally, energy flux can be used as a universal currency of ecosystem functioning that can be compared across different ecosystem types (terrestrial, aquatic–freshwater or marine) providing several functions that would otherwise be hard, if not impossible, to compare (Barnes et al., 2018; Odum, 1968). Additionally, the approach allows the quantification of processes that are usually hard to measure, such as, for example, above‐belowground interactions (Jochum & Eisenhauer, 2021). For example, many underground processes or the herbivory of sucking insect herbivores are hard to quantify with common methods. Instead, such process rates can be inferred from calculations of energy flux between the relevant species in the system, for example, belowground herbivores and their resources, or sucking herbivores and their resources (Barnes et al., 2020). When interested in such hard‐to‐assess processes, in ecosystem multifunctionality, or simply many different functions carried out by a specific target community, it may well be easier to calculate energy fluxes rather than measuring the desired processes directly. This can be done by assessing measurable attributes of the community (such as abundances and body masses) and using these to calculate energy fluxes. Moreover, given that investigators often measure proxies of the truly desired ecosystem processes (e.g., chewing‐herbivore damage as a proxy of overall herbivory), calculated energy fluxes are not necessarily less accurate than direct measurements of such proxies.

Energy‐flux calculation is based on the mere presence of the organisms, which implies their energy consumption in that system (they are alive, so their minimum energy demand must be met). As such, quantifying the present community of organisms and their interaction network allows the calculation of the energy flux through the community that is needed to maintain the energy demand they have for being alive. Calculating energy flux through ecological communities requires a combination of information on the focal community (Moore & de Ruiter, 2012), such as the organisms it comprises and their trophic interactions, their body mass, metabolic demand, network topology, feeding preferences, and trophic efficiency. While this may sound like very detailed, hard‐to‐obtain information, different levels of resolution are possible to obtain and consider for each of these aspects. For most aspects, there are reliable literature‐based parameters to calculate them based on relatively simple measurements. Here, we provide guidance on the sources of such parameters and what needs to be considered when using this kind of information for energy‐flux calculations.

The complexity and importance of assessing the flow of energy through ecological networks has been recognized for thousands of years (Moore & de Ruiter, 2012). The underlying theoretical foundation of energy flux based on both food‐web theory and biodiversity‐ecosystem functioning theory has been discussed (Barnes et al., 2018; de Ruiter et al., 1993; Elton, 1927; Hunt et al., 1987; Lindeman, 1942; Odum, 1968; O'Neill, 1969; Paine, 1980), and there are various example studies of how energy flux can be used to study important ecological questions (Barnes et al., 2014; Neutel & Thorne, 2014; Schwarz et al., 2017). There are methodological papers providing software to efficiently and effortlessly compute energy flux once the necessary data are available (Gauzens et al., 2018; Pauly et al., 2000; Ulanowicz, 2004a). However, the methodological details and decisions along the way towards successfully calculating energy flux can be challenging and may hinder progress in this field. This is particularly the case for those new to the topic, or those with challenging or unusual projects, for example, projects focusing on ecosystem types or taxa receiving comparatively little attention so that the necessary data on various aspects (metabolism, topology, and preferences) will be hard to find. Previous reports have listed the multiple steps towards calculating energy flux (Barnes et al., 2018) but, given their scope, could not comprehensively answer the question of how to obtain the necessary data or make informed decisions along the way. Most of the steps either require some in‐depth knowledge of the target system and community and/or some guidance on where to find the respective information (Table 1). However, to date, a comprehensive manual of how to address these challenges and a general introduction and critical discussion of the single aspects needed to calculate energy flux is lacking.

**TABLE 1:** Overview over the single steps involved in calculating energy flux with the adapted food‐web energetics approach

Step	Question	Options (how to get it)	Status	Minimum requirements	Example references
1. Community assessment	Who is there and how much?	Sample, assemble based on literature information.	Essential	Overall biomass and relative abundance (alternatively, only identity of trophic nodes and metabolic losses measured for each one). Taxonomic/functional resolution depends on the topological resolution (below).	Sousa et al. (2019), Decaëns (2021)
2. Energy loss – metabolic demand	What energy do they need to sustain themselves?	Measure (rarely possible) or estimate based on literature data or regressions.	Essential	Metabolic demand of the present organisms. Literature regressions based on organism type, body mass and temperature well available.	Brown et al. (2004), Hillebrand et al. (2009), Ehnes et al. (2011)
3. Body mass	How heavy are they?	Measure or infer from literature.	Useful for metabolic rates and passive preferences (relative resource availability)	None. Helpful for several steps (see Table 2).	Sohlström et al. (2018), Ruiz‐Lupión et al. (2019), Ärje et al. (2020)
4. Topology	Who eats whom?	Assess or assemble based on available information (feeding type, functional traits).	Essential	A simple food chain with at least two nodes.	Gravel et al. (2013), Eitzinger et al. (2019), Hines et al. (2019), Sousa et al. (2019)
5. Preferences	Relative consumption of resources?	Assess or infer based on relative resource availability, or functional traits.	Essential	Either assume all resources are consumed equally, or assume passive (density dependent) preferences, or provide additional information on active consumer preferences.	Gauzens et al. (2021), Pollierer and Scheu (2021)
6. Assimilation efficiency	Fraction of consumed energy available to consumer?	Assess or take from literature.	Essential	Resource type required (animal, plant, detritus, etc.). Available from the literature.	See Table 3

Note

For each step, the table outlines what the step is necessary for (Question), what are the options for achieving the required input information (Options), whether this step/information is absolutely necessary (status), what are the minimum requirements, and it provides example references helping with the decisions or pointing towards methods and literature resources to infer the input information. This table was inspired by figures in Jochum and Eisenhauer (2021) and Barnes et al. (2018).

This paper aims to fill this gap by providing a step‐by‐step explanation of the (most) critical steps towards the calculation of energy flux in complex systems and discussing challenges and research frontiers for this subject. We aim to cover a set of default steps that people will have to take in order to successfully calculate energy flux based on their community data. For each of the steps, we provide ecological background knowledge on why this is important and what we can do to get the most out of our energy‐flux calculation. We use the *fluxweb* R package and follow the adapted food‐web energetics approach of calculating energy flux, which bases flux calculations on the energy demand rather than biomass turnover of food‐web nodes (Barnes et al., 2018; for details see Section 2). Note that there are other ways to calculate energy flux in ecological communities, for example focusing on biomass turnover and mortality rates (Buzhdygan et al., 2020; de Ruiter et al., 1993; Hunt et al., 1987; Moore & de Ruiter, 2012), but *fluxweb* also covers these (Gauzens et al., 2018). While this paper focuses on one specific approach, many aspects will be just as important for a biomass‐turnover centered view. Finally, we highlight promising frontiers for future research on using energy flux for community and ecosystem ecology. With this paper, we aim to encourage fellow community and ecosystem‐ecologists to incorporate energy‐flux assessments into their research. Our goal is to help them get started on this subject that, initially, might seem very complex, but, once successfully adopted, will open up a whole suite of additional questions that can be answered about their target systems.

## 2 PRACTICAL CONSIDERATIONS

Several methods have been proposed to study energy flux through trophic networks (Pauly et al., 2000; Reuman & Cohen, 2005; Ulanowicz, 2004b). While these have been discussed before (Barnes et al., 2018), we will first explain why we focus on the adapted food‐web energetics approach here. Subsequently, we will introduce the reader to the general framework of calculating energy flux. The proposed methods to calculate energy flux mainly differ in their assumptions and the level of ecological organization (aggregation) at which certain parameter values are obtained and applied (species, ontogenetic development levels, body‐size groups, trophic levels). The food web energetics approach (Barnes et al., 2014; de Ruiter et al., 1993; Gauzens et al., 2018; Hunt et al., 1987; Moore & de Ruiter, 2012; O'Neill, 1969) is based on biomass stocks, ecological efficiencies (assimilation and production), natural death rates, and predation rates. A few decades ago, it was originally used to assess nitrogen and carbon mineralization rates through decomposition processes in soil‐food webs of different management (de Ruiter et al., 1993; Hunt et al., 1987). Later, the approach was adapted (i.e., “adapted food‐web energetics approach”) to calculate energy flux based on energy demand (metabolism) of the sampled community, rather than biomass stocks (hereafter metabolism‐ vs. biomass‐centered approaches), in combination with assimilation efficiency and loss to predation (Barnes et al., 2014, 2018). While the overall concept and framework of assessing energy flux is based on previous work, the main advantage of this adapted method is that it additionally takes the body‐size structure of the organisms, environmental temperature, and taxonomy into account—aspects that heavily influence energy flux because of their impact on organism metabolic rates—their energy demand (Barnes et al., 2018). This is because the energy demand of a given biomass of organisms varies with body‐size structure and temperature (smaller body size and higher temperatures increase energy demands, Brown et al., 2004). Thus, a metabolism‐centered view better enables us to take the impact of these biotic and abiotic drivers on energy flux into account. Moreover, as there already is a wealth of information on how organism energy demand changes with body size and temperature, these recent advances simplify the estimation of the parameters needed for energy‐flux calculations. The development of the *fluxweb* R package (Gauzens et al., 2018) has improved the accessibility of energy‐flux calculations (for both biomass‐ or metabolism‐centered investigations), so that we will relate our description of the single steps of energy‐flux calculations to how this can be achieved in *fluxweb* (details in Table 1 and the Appendix S1, full details of package functionalities are described in Gauzens et al., 2018).

**FIGURE 1:**
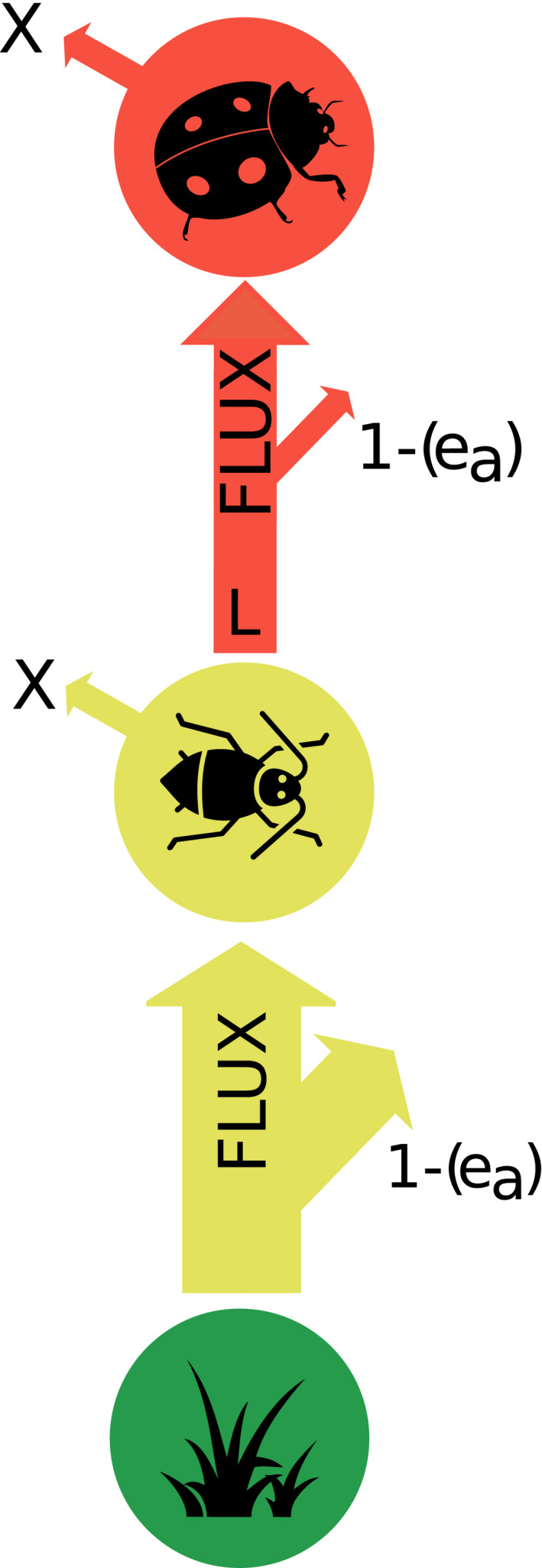
Calculating energy flux following the adapted food‐web energetics approach (Barnes et al., 2014, 2018). Energy flux along a trophic food chain from resources to highest‐level consumers is calculated with Equation (1) by taking into account metabolic demand by the consumer (*X*), assimilation efficiency (*e*_a_) and, for non‐top nodes, loss due to consumption by higher trophic levels (*L*). If calculating energy flux by hand, we would start with the flux between the highest‐level nodes in order to subsequently enable including loss to consumption (*L*) for all but the top node. Small arrows indicate “loss” due to energy consumption (metabolic demand, *X*) and efficiency losses due to assimilation efficiency (what is lost is 1−*e*_a_ * the flux out of the resource node). Predator node in red, herbivore node in yellow, plant node in green. For icon sources, please refer to acknowledgements

To calculate energy flux through a food chain or food web following the adapted food‐web energetics approach (Figure 1), we need information on the focal community of organisms forming this trophic structure. This includes information on who comprises the community (trophic identity of organisms), the energetic demand of the organisms (metabolic rates), who eats whom (network topology) to which extent (preferences), and how much of the consumed energy can be used by the consumer (assimilation efficiency; Table 1). Each of these aspects represents a gradient such that there is a minimum requirement of input information to calculate energy flux (e.g., low resolution, literature‐derived information on assimilation efficiencies), but higher‐resolution information (e.g., high‐resolution data, individual‐level data, assimilation efficiency measured for the specific species relevant in the study context) can always be applied. As there is little information on how the resolution of data and shifting along these gradients affect the outcome of energy‐flux calculations, we suggest to use more‐detailed information where possible, but of course this will usually be constrained by the available time, work force (person hours/team size), and project funds. The details of shifting along these gradients of varying resolution will be discussed in the specific chapters below.

In short, to calculate energy flux along a food chain, we start from the top node (terminal compartment sensu O'Neill, 1969) and assess how much energy is needed there. Given the assumption of conservation of matter and energy flow (Moore & de Ruiter, 2012), this energy then needs to come out of the next‐lower level. As such, it is assumed that the energy demand of organisms at a given level must be met by the energy intake of that level, that is, there is an equilibrium of in‐ and outflux (Barnes et al., 2018; O'Neill, 1969). Because of ecological efficiencies (e.g., consumers cannot use all consumed energy for respiration or to produce biomass—some of it is excreted), in order to fulfill its energy demands, every node needs to consume more energy from the next‐lower node(s) than it actually requires because there are costs of trophic energy transmission. The energy consumed from the lower‐level node is then treated as energy loss from this node. This loss to consumption is then added to the energy demand of the resource node itself to represent the joint energy loss of this node that needs to be compensated by the next‐lower level and so on. As such, the predator in Figure 1 only has metabolic losses (*X*), while its resource, the herbivore, has both a metabolic loss and loss to consumption (*L*).

A single flux is calculated as

(1) $$F=\frac{1}{e_{\text{a}}}\cdot (X+L)$$

where *F* is flux out of the resource node, *e_a_* is assimilation efficiency, *X* is metabolic demand, and *L* is loss to consumption of higher‐level nodes (Barnes et al., 2014, 2018).

Several of the following considerations are dependent on the type of project that is carried out. Depending on whether the focal project is based on existing data from a previous sampling campaign or a new project is planned with the freedom to make decisions on which aspects to measure or sample, we have different options. If the calculation of energy flux is based on existing data, certain aspects will be fixed (e.g., the level of taxonomic/functional identification of organisms, or the area/volume that has been sampled for different taxa), but others can be complemented using literature data (e.g., assimilation efficiency, feeding preferences, or network topology). If designing a project from scratch, investigators have the opportunity to decide what level of precision and resolution they would like to and are able to achieve for which parameter in order to answer their specific research questions. Even for data‐synthesis studies, additional measurements can sometimes be taken to enable a different topological resolution or more‐detailed information on physiological or ecological parameters. As such, we could for example assess individual body masses of organisms to gain a better idea of community‐size structure. Moreover, we could more closely inspect certain taxa or samples to validate their trophic role in the community, for example, using food‐choice experiments or molecular methods to assess feeding links and preferences (e.g., molecular gut‐content analysis, fatty acid analysis, stable‐isotope analysis, see Section 3.3.1).

### 2.1 Community assessment

**FIGURE 2:**
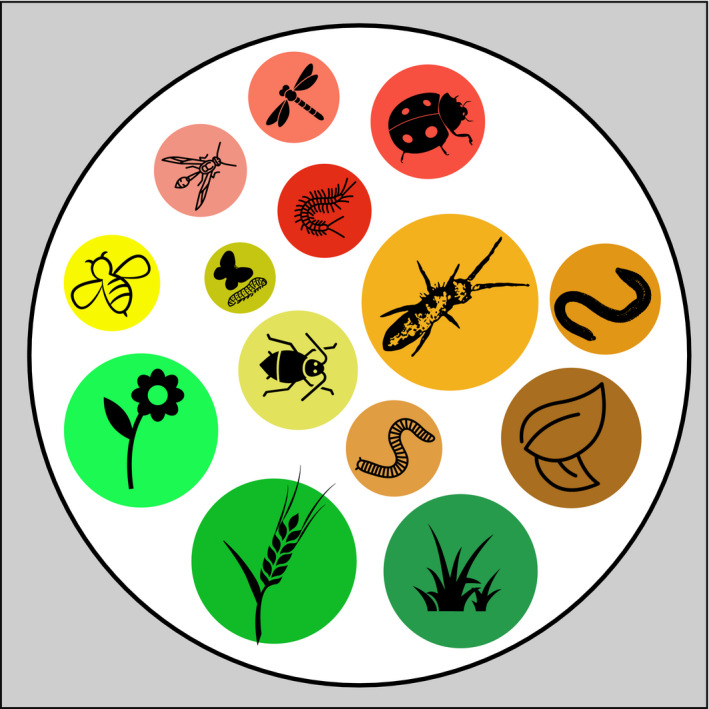
Community assessment. In order to calculate energy flux, we need to assess biomass densities (circle diameter) of all organisms comprising the trophic community, that is, all organisms feeding on each other. One approach to achieve this, is to sample a specific subcommunity present in a given stratum per area (terrestrial) or volume (aquatic). This could, for example, be the aboveground arthropod community in a grassland patch of a given area. Organisms are then sorted into trophic feeding guilds or even species to build nodes of the food web to base the energy‐flux calculation on. Here, predators in red, herbivores in yellow, detritivores in orange. This community is then considered together with their basal resources (here: plants in green and detritus in brown). For icon sources, please refer to acknowledgements

First of all, we need to know which organisms are present and with what abundance or biomass (Figure 2, Table 1). This information is necessary, as without knowing the sheer amount of organisms present and what trophic role they play in the community, we cannot assess how much energy they will require to survive, let alone how much energy will flow through the trophic network to sustain all these organisms while also accounting for energetic losses. We will go through the details of what information we would ideally collect (sample or retrieve from original data collectors or the literature) below. The minimum information will be some kind of overall biomass and some idea of the relative abundance/importance of functional feeding groups. Obviously, more‐detailed information will provide deeper and more‐nuanced insights into energy flux through the community and the related ecosystem processes or services. As such, the closer we can get to individual‐level data on body mass, metabolism, feeding preferences, or assimilation efficiency, the more detailed the retrieved information. However, in some cases, it is still unclear to what extent an increase in the resolution of input information will have an impact on the estimated fluxes. For more detail on the effect of topological aggregation or the impact of using metabolic scaling regressions, please see below. In any case, the focal research question should ideally define the level of resolution and detail in calculating energy flux.

#### 2.1.1 A matter of scale

One aspect that is important to consider before attempting to plan a new sampling campaign or calculate energy flux from existing data is spatial scale and its relationship with the study objectives. This is important as it will define what food‐web perspective we will adopt to answer the research question and, subsequently, how to perform the community assessment or which of the available data to use. Cohen (1978) identified three different categories of food‐web descriptions: the community‐food web, the source food web, and the sink food web. In short, a community‐food web approach is defined by a habitat and contains the species and interactions within this habitat. A source food web approach is based on a (set of) resource(s) at the base and then includes all feeding interactions up to top predators. A sink food web defines a sink, a (top) predator (level), and then includes all their resources and the resources' resources down to the basal resources. All of these approaches are constrained, as they exclude linkages that are also related to the species they comprise, such as interactions across habitat borders (community approach), interactions with additional resources (source) and consumers (sink) not linked to the focal source or sink (Moore & de Ruiter, 2012). This means that calculated energy‐flux quantities are always related to this choice of approach and will necessarily over‐ or underestimate the true flux through these systems per area.

When using energy flux to assess ecosystem processes or functions, for example, in plot‐based studies, we are usually interested in the rate of functioning per area or volume in space. As such, it would be ideal to take a community‐food web perspective (sensu Cohen, 1978) and assess energy flux per area or volume (we will speak of area below, but mean both area or volume, depending on the study context). We should thus keep in mind that any interactions with organisms only partly foraging within this area (e.g., larger‐bodied predators that forage over larger spatial extents) are often ignored. If our community contains species that operate over larger spatial scales than all others, these should either be excluded, or dealt with by (a) estimating which part of their energy demand is provided by the study area and then (b) including them in our network topology with that fraction of their energy demand. To meaningfully assess energy flux per area, we therefore need to assess the organism community in that area and everything that, in terms of energy transfer, belongs to this community (even if only partly) and thus impacts energy flux at that spatial scale. Given the impact of high‐trophic level organisms on overall energy flux (due to energetic losses along the food chain—see assimilation efficiency—top‐level energy requirements are only a fraction of the energy that needs to be taken out of the basal resources to maintain these high‐trophic level organisms) (top‐)predators are very important in this aspect. We do not necessarily have to assess all organism types on the same spatial scale, but they need to be scaled to a spatial extent defining their energetic relatedness. A standard approach can be to scale all organism assessments to the same spatial extent, exclude organisms likely operating at much larger extents, and assume that this is the most relevant community for the desired spatial extent of the focal ecosystem‐process assessment. It should be noted that this is not a problem specific to energy‐flux calculations but to most of community ecology. It is important to carefully think about what defines a community and which interactions are kept out of sight by choosing one of the perspectives.

Irrespective of the chosen level of resolution, a frequent challenge is getting all community data to the *same spatial and temporal scale* (see Section 2.7.2). To meaningfully calculate energy flux within a community, it is important that all organisms (species, trophic groups) are assessed for the same spatial scale, thus including those organisms that are feeding on each other—using one scale is basically a workaround for doing this as long as organisms are not too mobile (if animals, e.g., fly in to feed and then leave again, snapshot‐sampling them just in a small area will not adequately capture their impact on the system). We thus need organism densities rather than just individual counts (abundance) or biomass per se.

### 2.2 Energy loss

Once the community of organisms has been sampled or the respective data assembled, we can start looking into energetic aspects. Energy is needed at each node to survive (metabolic demand). Additionally, all but the terminal (highest trophic) node(s) have losses due to consumption. We can look at all of this in terms of biomass turnover and include natural death rates and predation rates, or we use the adapted food‐web energetics approach and view it all through the lens of metabolism (*fluxweb* offers both, but here we focus on the latter). If we take this approach, we need to include the node's own energetic demand (metabolic rate), loss to consumption, and assimilative losses (efficiency) when transferring energy from one node to the next. It should be noted that what we calculate is the minimum energy flux required to keep the community alive (see below). Here, we focus on the adapted food‐web energetics approach, but the concept is the same no matter what *fluxweb*‐supported approach we take: At each node, there are losses and gains and these need to be balanced out.

#### 2.2.1 Metabolic demand

**FIGURE 3:**
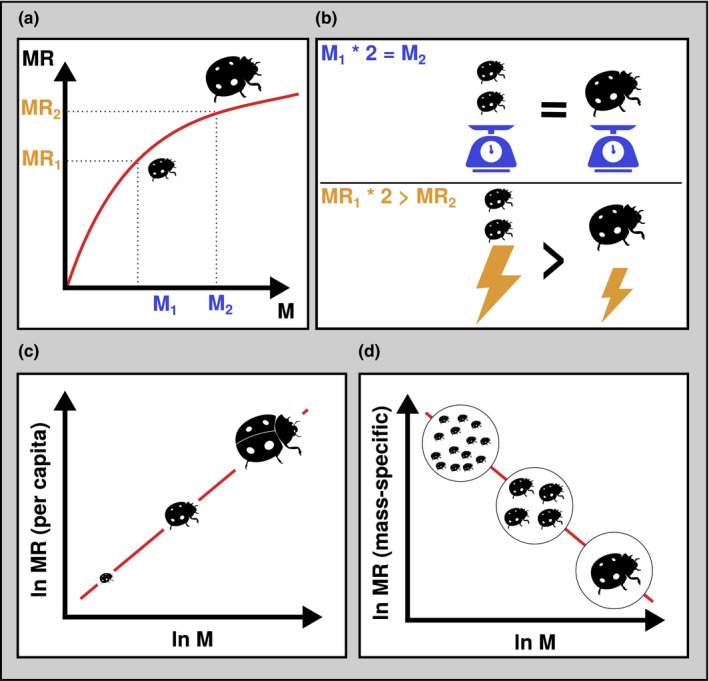
Metabolic rate (MR) is the rate of “energy uptake, transformation and allocation” (Brown et al. (2004), metabolic theory of ecology). It is directly related to each organism's energy demand and, as the “fundamental biological rate” (Brown et al., 2004) determines many other physiological and ecological rates of an organism. It scales with body mass (M) and temperature (not shown here) according to a power law (a). (a) Nonlinear relationship between body mass and metabolic rate. Using two example animals from (a) (with masses M1 and M2 and metabolic rates MR1 and MR2), (b) illustrates how body masses and metabolic rates are not related by the same factor. This means that, while per‐capita metabolic rate increases with body mass, mass‐specific metabolic rate decreases. In other words, the metabolic demand of one large individual is lower than the joint metabolic demand of several smaller individuals with the same cumulative biomass: Per‐capita metabolic rate increases with body mass (M) following a power law (c), but mass‐specific metabolic rate declines with body mass (d). All else being equal (environment, taxonomy, etc.), the metabolic demand of a large individual is higher than that of a small individual (c). However, the joint metabolic demand of a group of small‐bodied individuals is higher than that of a smaller number of larger‐bodied individuals with the same cumulative biomass (d). For icon sources, please refer to acknowledgements

All nodes in our food web have an energetic demand (*X* in Figure 1). For those nodes with higher‐level consumers above them, their overall loss is the sum of their own energetic demand and the loss to consumption (accounting for both ecological efficiency and the consumer's energetic demand). Within the food‐web energetics approach, we use metabolic rate as the measurement of an organism's energetic demand. Metabolic rate (Figure 3) is the rate at which energy and materials are taken up, transformed and allocated (Brown et al., 2004). For heterotrophs, metabolic rate equals respiration rate; for autotrophs, it is equal to the rate of photosynthesis (Brown et al., 2004). It can be measured by measuring the rate of autotroph carbon dioxide uptake or heterotroph oxygen consumption.

The metabolic theory of ecology (Brown et al., 2004) proposes that metabolic rate is driven by body size, temperature, and stoichiometry, and describes its power to control fundamental ecological processes related to survival, growth, and reproduction across levels of ecological organization (individual to ecosystem). Its dependence on body size, temperature, and other biotic and abiotic aspects is of fundamental importance as these aspects will ideally be taken into account to estimate metabolic rates as exactly as possible. We will focus on a few key aspects here.

First, the body size of an organism is strongly related to how much energy it needs. Generally, larger organisms need more energy than smaller ones (Figure 3c). However, the relationship between body mass and metabolic rate is not linear, but defined by a power law, such that several smaller individuals of the same cumulative biomass as one larger organism will need a higher amount of energy than the large individual (Figure 3a,b,d). All else being equal (temperature, taxonomic identity, etc.), the mass‐specific metabolic rate decreases with body mass, while per‐capita rates increase. Second, metabolic rate increases with temperature. The warmer it is, the more energy is required. Therefore, ambient temperature will be one key abiotic aspect that users will need to obtain or estimate (e.g., based on global temperature datasets) for calculating energy flux. Additionally, both the temperature and body mass dependence of metabolic rates differ between taxonomic groups (Brown et al., 2004; Ehnes et al., 2011). Depending on the focal study system, other aspects will additionally impact metabolic rates. For example, Jeyasingh (2007) found different allometric scaling exponents based on whether consumers were fed stoichiometrically balanced vs. imbalanced diets. In summary, body size and temperature are key aspects to take into account when estimating metabolic demands.

There are different ways of obtaining metabolic demand for your study organisms. Most importantly, it can either be measured, taken from literature reports (e.g., as per‐individual average essentially ignoring body mass), or calculated based on literature‐derived relationships with its main driving variables (body mass and temperature). In the first case, respiration of individuals or trophic groups can be measured (see example from Lefcheck & Duffy, 2015 in worked example of Barnes et al., 2018). This can either be done in situ (but it will often be hard to isolate certain trophic groups or species to obtain a measurement of just their metabolic demand) or in the laboratory. In most cases, however, measuring metabolic demand will not be feasible. Instead, given its strong relationships with body size, temperature and taxonomy, it is relatively easy to calculate metabolic demand based on these variables (see example for arthropod metabolic rates in Box 1). Depending on the given trophic groups and ecosystem, it might be easier to find literature values for respiration rather than finding adequate regressions for body mass/temperature ‐ metabolism conversions (e.g., for nematodes see http://nemaplex.ucdavis.edu/Ecology/EcophysiologyParms/EcoParameterMenu.html), but for most taxa general metabolic‐theory regressions (Brown et al., 2004) will be available. Here, we will focus on calculating metabolic rates based on the above‐described well‐established relationships with body mass, temperature, and taxonomy. Before we delve into this, a few words of caution: First, what is measured and provided in the literature are often reports of the basal (also: resting or standard) metabolic rate of animals in a resting state. The active or field metabolic rate, over longer periods of time and under realistic conditions, is typically a multiple of this basal metabolic rate (Savage, Gillooly, Woodruff, et al., 2004). This should be kept in mind, as it means that the energy flux we calculate based on basal metabolic rates is likely to be the absolute minimum of energy flux to sustain the given community of organisms. Second, although metabolic scaling theory is well developed (Brown et al., 2004) and its predictions have found support in various taxa and ecosystems (Padfield et al., 2018; Riveros & Enquist, 2011), there is still some discussion about the exact scaling exponents and their causes (Savage, Gillooly, Brown, et al., 2004). Several studies have furthermore documented important additional aspects providing complementary information to pure metabolic theory, for example, phylogeny (Ehnes et al., 2011) or consumer‐resource stoichiometric mismatches (Hillebrand et al., 2009). Additionally, when using metabolic theory to estimate metabolic rates based on body mass and temperature, the resolution of input information on these two parameters obviously has a big impact on the result. Thus, investigators should carefully consider what metabolic information might be directly measured or which input information is available for their focal system and community before simply using the easiest‐available regressions on average temperatures and body masses. In this vein, individual‐level information on body size is a big advantage when using metabolic scaling regressions (Barnes et al., 2014; Padfield et al., 2018) and every step towards this direction (e.g., not assessing all, but many individuals, instead of just using average masses based on measuring very few individuals) is a plus. Most importantly, however, it is important to acknowledge and discuss the limitations and uncertainties that are related to using such regressions.

When using the adapted food‐web energetics approach, one does not require metabolic information on the basal resources, such as detritus or nutrient supply. For example, when using plants or detritus as basal nodes (see example in Figure 1), their metabolism and efficiency of energy transformation for their energy intake are not required, unless we wish to quantify their energy intake and use their resources as the true base of the food web. This simply means that our energy‐flux assessment does not include the energy flowing into these basal nodes (or lost when converting energy due to converting efficiencies), but only that energy flowing out of this basal level, as defined by the energy demand of the first consumer level and the respective assimilation efficiency.

*In fluxweb*, metabolic rates are applied as loss terms for each node and supplied to the *fluxing* function as argument *losses* (Gauzens et al., 2018). Note that if argument *bioms.losses* is set to *TRUE* (default behavior), then the *fluxing* function expects losses to be provided on per gram biomass and will consequently multiply these losses by the respective biomass of the node. If losses have, for example, been calculated as metabolic rates on a per‐capita basis, then these single metabolic rates need to be summed up for each node to obtain the full metabolic demand of each node. In this case, argument *bioms.losses* needs to be set to *FALSE* and the *fluxing* function will expect losses to be provided on the node level. Please note that fluxweb is flexible in regard to input units and that the output units naturally depend on the input (Gauzens et al., 2018). If, for example, metabolic rates are entered in W, then flux is also estimated in W. What is important is that unit use is coherent throughout using the package: If, for example, losses are provided per mg fresh biomass, then biomass needs to also be provided in mg fresh mass.

BOX 1 **Calculating arthropod metabolic rates from body mass and temperature**

For most projects, metabolic rates can be calculated based on universal metabolic scaling relationships (Brown et al., 2004). However, for several taxa, there are more‐detailed regressions available. For example, metabolic rates for terrestrial invertebrates can be calculated using taxon‐specific regressions from Ehnes et al. (2011). These regressions are based on body mass and temperature. Ehnes et al. (2011) group Collembola with insects, but provide specific metabolic rate regressions for Oribatida, Prostigmata, and Mesostigmata. However, the raw data are published and thus other groupings of interest can manually be processed (Ehnes et al., 2011). Note that when using such regressions, it is of imperative importance to carefully check units and other details of required inputs and delivered outputs (see Section 2.7.1 for more detail). Some sources provide metabolic rates on a per‐unit‐biomass basis, others provide per‐capita metabolic rates for individuals of a given body mass. The metabolic rate output units will define our energy‐flux output units if they are not further transformed (e.g., to provide flux in kg fresh mass per hectare per year, see Barnes et al., 2014). Please refer to Appendix S1: Section 2.1, for R‐code on this calculation.

### 2.3 Body mass

The body mass of an organism is related to its physiology and ecology in many ways (Brown et al., 2004; Damuth, 1981; Kleiber, 1947; Peters, 1983), and a lot of research focuses on how body size affects ecology from individuals to ecosystems (e.g., Kalinkat et al., 2015). Several of these aspects make body masses very interesting for the purpose of energy‐flux calculation (Table 2). As we have seen, body mass can be used to estimate metabolic rates, but it can also provide us with biomass estimates that are helpful to scale passive feeding preferences (see below). Furthermore, the relative body mass of consumers and their prey (body mass ratio) has long been recognized as a driver of ecological interactions (Brose et al., 2019; Gravel et al., 2013; Schneider et al., 2012) and can, for example, be used to infer feeding preferences and network topology (set feeding links, see e.g., Hines et al., 2019).

**TABLE 2:** How organism body mass affects energy flux, split into the different aspects of energy‐flux calculation that we introduce

Affected aspect	Explanation	Example references
Metabolic demand	Per‐capita metabolic rate increases, per‐unit‐biomass metabolic rate decreases with body mass.	Brown et al. (2004)
Topology	Predator–prey body mass ratios affect who feeds on whom in ecological networks.	Brose et al. (2019)
Preferences	Predator–prey body mass ratios determine the relative consumption of different prey items. Body mass can also be used to assess biomass densities of organisms in our communities, which can be used to set passive preferences (see below).	Schneider et al. (2012), Gauzens et al. (2018)

#### 2.3.1 Assessing body mass

Body mass is often indirectly assessed by measuring bulk biomass and dividing it by the number of individuals (abundance), or such averaged values are instead obtained from functional‐trait measurements or databases. These options have the disadvantage that they do not provide individual‐level data, more specifically, they do not provide any information on intraspecific variation in body size. As we have seen above, metabolic rate changes with body mass, and mass‐specific metabolic rate decreases with body mass. This means, an individual of a given body mass needs less energy than several smaller individuals with the same cumulative biomass. Consequently, the body size structure of a population (or of individuals within a trophic node) thus impacts the energy demand of the population. Therefore, as long as the actual respiration of a population will not be directly measured, individual‐level data on body mass is ideal in order to assess energetic demand with respect to the nonlinear relationship between individual body mass and energy demand.

Of course, body mass can, in theory, be measured for each individual. However, this is very time and labor intensive and, especially for small‐bodied animals, problematic as the measurement of, for example, small arthropods requires temperature‐ and moisture‐regulated weighing rooms and precision scales that will not necessarily be available. Sometimes, measuring body mass will not be possible if the organisms are not accessible, such as for projects synthesizing data from databases. However, in many cases, there will be options to obtain photographs or video footage of animals that can be used to obtain length estimates of whole bodies or body parts. This approach could also be used where animals cannot or do not need to be sampled, for example, because they are meant to stay relatively undisturbed. The resulting analysis of images to obtain body mass estimates is nondestructive and effective (Llopis‐Belenguer et al., 2018).

Depending on the study system and research question, it might be reasonable and feasible to obtain a decent level of body mass information by measuring individual body parts (overall length or selected body parts) and using literature‐derived regressions to calculate individual body mass from these measurements (Ruiz‐Lupión et al., 2019; Sohlström et al., 2018). Please refer to Box 2 for details on length‐mass regressions for terrestrial arthropods. For different taxa, there generally are different standard ways of measuring a body part (head capsule width, hind‐leg length, carapace width, etc.) or the length of the whole animal and relating that to body mass via a regression that has been fed with length and mass data of, ideally, many individuals covering the typical length range for the given taxon. Here, we will focus on terrestrial arthropods, but similar regressions are available for other taxa including, for example, fish (fishbase database, https://www.fishbase.de/manual/fishbasethe_length_weight_table.htm), amphibians (Santini et al., 2018), and reptiles (Feldman & Meiri, 2013). Combined automated, image‐based identification and biomass estimation will likely improve over the coming years so that estimating individual‐level body mass data for large numbers of specimens will become more broadly available (Ärje et al., 2020).

BOX 2 **Calculating arthropod fresh body mass from body length**

For arthropods, for example, the literature holds a decent collection of length‐mass regressions (Gruner, 2003; Mercer et al., 2001; Ruiz‐Lupión et al., 2019; Sohlström et al., 2018). When choosing length‐mass regressions for your study, keep in mind that the productivity of the target system (Ruiz‐Lupión et al., 2019) and the geographic region (Sohlström et al., 2018) affect length–mass relationships and should thus be taken into account, where possible. Furthermore, several studies provide regressions combining more than one measured body dimension (Gruner, 2003; Sohlström et al., 2018), for example, body length and width, and show that these models have better fit than single‐morphological predictor models (Sohlström et al., 2018). Thus, it might be reasonable to measure more than one dimension for your animals. This might be particularly helpful when taxonomic information on the study organisms is lacking or when no regressions for the required taxa are available. We provide example R code to calculate body masses for terrestrial macro‐ and soil mesofauna based on literature‐derived regressions. Specifically, these examples include regressions from (Sohlström & Jochum, 2021; Sohlström et al., 2018) for several macrofauna taxa, for just length or both body length and width, and for both temperate and tropical arthropods. Soil‐mesofauna body masses can, for example, be calculated using regressions from (Mercer et al., 2001). Please refer to Appendix S1: Section 3.1, for R‐code on this calculation.

One aspect to keep in mind here is that what we typically need is live/fresh body mass, because this is what most scaling relationships use to calculate metabolic demand. This seems obvious for people working in, for example, movement ecology or with physiological rates, but other researcher groups tend to always use dry mass for their research (e.g., for stoichiometry or nutrient content), and thus a lot of what is available, for example, for length–mass regressions comprises only dry masses. There are different ways of converting dry masses to fresh masses, such as another set of regressions (Mercer et al., 2001) or simply using rough conversion factors. However, ideally, fresh masses are directly calculated by using suitable regressions.

When using *fluxweb* to calculate energy flux, body masses are only used indirectly, for example, via estimating losses due to metabolism, to calculate biomasses that can then be used to set passive preferences, and to initially set up network topology and feeding preferences (Gauzens et al., 2018, Table 2 for further detail).

### 2.4 Food‐web topology

**FIGURE 4:**
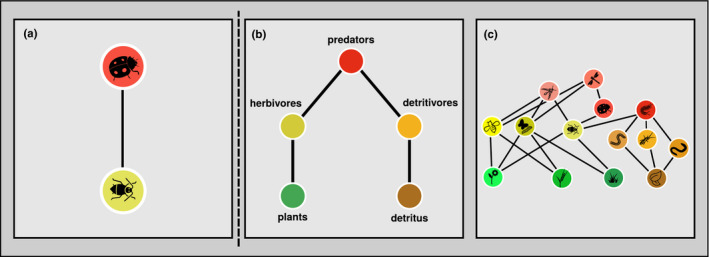
Topology. Food‐web topology is the arrangement of elements (nodes—trophic “species”, and links—feeding interactions) in a trophic network. Depicted are three different topologies. (a) Example of a two‐node food chain where a lady‐beetle population feeds on an aphid population, for example, in a simplified experimental setting. (b and c) could represent the same underlying community with different resolution, where (c) (species level) has a higher resolution than (b) (functional feeding guilds). For icon sources, please refer to acknowledgements

In order to calculate energy flux through a trophic network comprising an ecological community, we need to know which pathways this energy flux takes in this particular community—that is, we need to know the food‐web topology, or, simply put, who eats whom (Figure 4). This knowledge is one of the most fundamental aspects of describing communities of organisms, and its scientific description is subject of research on food webs. The food web consists of trophic species (nodes) and their feeding interactions (links) which, together, form the topology of the food web. In its simplest form, a food web represents a food chain of several nodes linked by single feeding interactions (see example in Figure 1). However, food webs can have thousands of nodes and even more links. For our purposes, food webs do not necessarily have to be resolved to species level. Early use of the adapted food‐web energetics approach has mostly made use of relatively broad functional feeding groups, such as predators, herbivores, and detritivores (Barnes et al., 2014) or more specific groups combining taxonomy with feeding types (Barnes et al., 2020; de Ruiter et al., 1993). This was at least in part driven by practical constraints as computational tools for the calculation of energy fluxes (e.g., the *fluxweb* R package) were not available and analytically solving certain food‐web structures such as trophic loops was challenging. Now, the computational tools are available, but we still know little about what impact different topological resolutions have on the resulting flux calculations.

That said, how do we obtain the information on food‐web topology for our energy‐flux calculation? There is a multitude of different approaches to assess or infer feeding links (Bartomeus et al., 2016; Cirtwill et al., 2019; Gravel et al., 2013; Hines et al., 2019). Which ones can be used, depends on the type of project and the desired or achievable resolution of the topology. The more information is available and the less well‐resolved the desired food‐web topology, the easier. In most cases, it should be possible to group organisms from a community sampling into coarse functional feeding groups, such as predators, herbivores, and detritivores, which can often be done based on taxonomy. For taxa reported to feed on several food resources, this can later be dealt with by setting preferences (see Section 2.5), but both links would be set in the topology. If we have to rely on available data on previous community assessments and the organism samples are not available or not usable anymore, what we can do strongly depends on what data is available. If we have, for example, species names, a lot can still be inferred by taxonomy. If we have abundances per feeding group, we can use this level of aggregation. If we can design our study from scratch, we are more flexible, but also have to decide what level of topology is both desirable and achievable. The more specific the community data, the more flexibility do we have in the resolution of the chosen topology. In such cases, we can even test the impact of varying topological resolution on our results.

Although specific information on how topological resolution (aggregation of organisms into nodes) affects energy‐flux calculations is sparse, we can use insights from related topics (aggregation impacts on food‐web structure) to gain an idea of what impact the chosen level of aggregation might have, how much aggregation would be acceptable, and how different types of aggregation may affect our results. Aggregation is an important factor in ecology and the potential issues resulting from it have been discussed (Buchkowski & Lindo, 2020; Gardner et al., 1982; Gauzens et al., 2013; Pinnegar et al., 2005). Two distinct types of aggregation are relevant here: serial and parallel aggregation. Parallel aggregation is the aggregation of two populations into, for example, a trophic level (Gardner et al., 1982)—in our case it could also be two different predator groups that we treat as one trophic node of predators. Serial aggregation occurs, where we group several adjacent components of a food chain into one node. For example, this would occur where we group second‐ and first‐order predators in one predator node. Gardner et al. (1982) found parallel aggregation to be a minor issue for simulations of ecological process rates through interaction networks as long as it aggregated over components with similar input and output rates. Serial aggregation was found to be more problematic, but also depended on how similar the aggregated components were in their ecological rates. A study looking at the effects of aggregating a marine food web in two steps from 41 to 27, and then to 16 compartments found system properties (connectance and system omnivory) and dynamic stability to be altered (Pinnegar et al., 2005). Another study looking at topological‐aggregation effects of predator foraging behavior and biomass on food‐web topology found that results, that is, the relationship between food‐web structure and ecosystem functioning, were preserved over a large proportion of the topological‐aggregation gradient (Gauzens et al., 2013). Another study looking at how “lumping” of trophic species affected C and N mineralization rates found that lumping effects depended on how similar the lumped species were in ecological efficiencies and their diet (Buchkowski & Lindo, 2020). Taken together, for our energy‐flux calculations, these findings indicate that grouping taxa with a similar trophic role (feeding on the same resources and being fed on by the same consumers; parallel aggregation) and ecological efficiencies (assimilation efficiency) might be rather unproblematic. Aggregating animals that actually feed on each other (serial aggregation) might be more problematic. It is important to note here that most of the available information is for aggregation effects on topological parameters and certain process rates, while information on aggregation impacts on energy flux and specifically the structure‐function link is still scarce. In addition, it should be noted that these conclusions only hold for aggregating topology. As discussed above, aggregating organisms of different body size for calculating metabolic demand should be avoided, as size‐metabolism relationships are not linear. Given this sparsity of information regarding aggregation impacts on energy flux, investigators should discuss the uncertainty introduced by using aggregated food webs and discuss the potential impact of this aggregation on their conclusions.

Whenever taking new measurements, we could, for example, make use of gut content analysis, digestive enzymes, fatty acids, and stable‐isotope analysis or combine several of these analyses to identify a multidimensional trophic niche (Potapov et al., 2020). Alternatively, in order to infer links for existing data, there is a number of aspects that could be taken into account. Previous studies have, for example, used a combination of literature‐derived information on feeding links (specific: taxa reported to feed on a specific species, or generalized: taxa reported to feed on all species in a taxon), trophic level, trait‐based rules (relying on, e.g., body size, trophic level, taxonomy, consumer biting force and resource toughness, and/or overlap in vertical stratification of taxa), and phylogeny (Brousseau et al., 2018; Hines et al., 2019; Laigle et al., 2017). Given the availability of stoichiometric data, links could also be inferred based on a minimized stoichiometric mismatch (Hillebrand et al., 2009). Traditional food‐web ecology has furthermore developed well‐established rules to set links in artificial networks used for example in modeling studies. These rules are based on observing natural food webs and then developing algorithms to set links in artificial networks that are supposed to closely mirror the structure of real food webs. These rules are traditionally heavily based on body size and are of varying complexity (Dunne, 2006). Recent research has shown that various consumer and resource traits differ in their impact on who feeds on whom in ecological networks (Brose et al., 2019). Thereafter, feeding links depended more heavily on predator traits, such as predator metabolic group or movement type, than on the equivalent prey traits.

Given the nature of the project and data availability, we need to define a desired topological resolution and then set the links. In the most simple case, this will just be the feeding interaction between two species known to have a trophic relationship (Figure 4a). In a low‐resolution (broad trophic feeding guild) example, this could be predators feeding on herbivores and detritivores who, in turn, feed on plants and detritus, respectively (Figure 4b). With increasing topological resolution, we will have to rely more heavily on functional traits of the organisms and / or specific measurements or available data on feeding interactions (Hines et al., 2019; Potapov et al., 2020) to set links for combined taxonomic and feeding guilds (e.g., fungivorous Collembola feeding on all fungi in soil, or herbivorous macroarthropods feeding on all plants available, de Ruiter et al., 1993; see also Barnes et al., 2020). If well‐resolved food‐web data are available (Figure 4c), this can simply be used either at the highest available resolution or looking at different levels of resolution (aggregation, e.g., comparing the same food web at different aggregation levels, Figure 4b,c) to ensure robustness of results. In a theoretical (e.g., simulation) context, we are of course most flexible as to what approach we use to set food‐web links and can choose between relatively simple (Dunne, 2006) or more‐refined rules, for example, based on functional traits (Brose et al., 2019; Hines et al., 2019).

Taken together, depending on the research question and data availability, there is a variety of approaches to assess or set food‐web links and build a network topology that can then be used in subsequent energy‐flux calculations. Basic functional traits of the coexisting organisms such as feeding type, body size, and movement type can already achieve a well‐defined food‐web topology. If desired, more basic rules can be complemented with more specific measurements on feeding interactions in a given community. These can also be very useful in setting preferences (see Section 2.5). It should be noted that, due to the relative novelty of a broader application of this energy‐flux approach, to our knowledge, there is no assessment of how much varying topological resolution drives energy‐flux results. Aside from some expected quantitative differences driven by varying topological resolution, there could also be qualitative differences, for example, depending on the proportions of generalists versus specialists in the focal community. We might expect more variation with topological resolution, if there are more specialists in the focal community, because specialist feeding cannot be accounted for in lower‐resolution networks. This could, for example, be more of an issue in terrestrial versus aquatic systems, because the latter are expected to have, in general, a higher proportion of generalist species (Shurin et al., 2006).

In *fluxweb*, topology is supplied as a matrix to the *fluxing* function via argument *mat*, with consumers in columns and resources in rows (Gauzens et al., 2018).

### 2.5 Preferences

**FIGURE 5:**
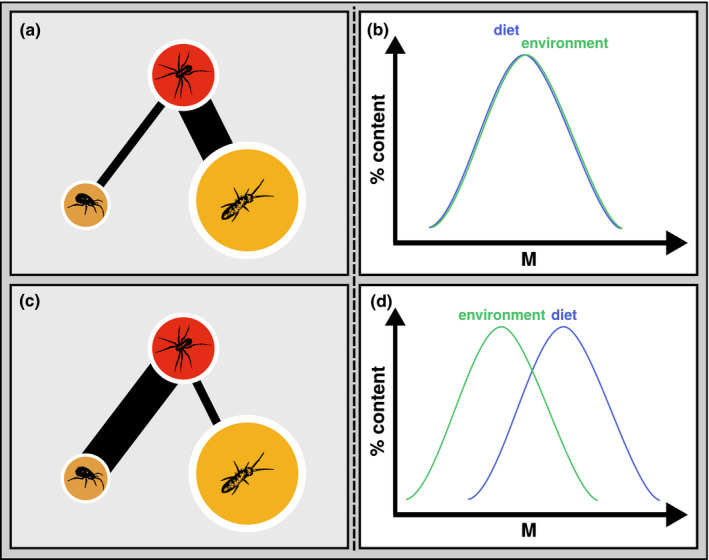
Preferences—passive (upper row, (a) and (b)) and active (lower row, (c) and (d)): Passive feeding preference (a) is illustrated by a consumer population (red node, centipede) feeding more heavily (link width) on more‐available prey (prey biomass of millipede and springtail populations depicted by node diameter). In this case (b), the distribution (% content) of prey in the environment (green) and in the consumer diet (blue) across prey body size (M) is equal. Active preference (c) is illustrated by the consumer population feeding more heavily on the less‐available prey (here: mites), for example, due to preferred predator–prey body size ratio or lower stoichiometric mismatch between predator and prey body tissue. Here (d), the distribution of prey body mass in the diet is clearly shifted towards the larger‐bodied prey compared to the distribution in the environment. Illustrations in (b) and (d) are motivated by Gauzens et al. (2021). For icon sources, please refer to acknowledgements

We have now established that the different organisms, or, more generally, consumer nodes in the trophic network of our community, consume different resources. The next topic we will cover is the consumers' feeding preferences (Figure 5). It is intuitively clear how unlikely it is that a consumer feeding on multiple resources does so with equal relative feeding intensity. Preferences may be active, that is, driven by consumer choice due to, for example, resource quality, size, defenses, or the ability of prey to escape or fight back (handling time). Additionally, consumers may be passively driven to consume different resources in different proportions simply based on the relative abundance / availability of these resources. Resource quality (defenses, stoichiometry, etc.) varies a lot between different autotroph resources (McGroddy et al., 2004; Sterner & Elser, 2002), but also for different heterotroph resources (Fagan et al., 2002; González et al., 2011; Martinson et al., 2008). Consequently, it will also vary in detrital resources (Martinson et al., 2008; McGroddy et al., 2004) even if this variation might be less extreme because plants resorb their nutrients before leaf abscission. However, it is not only resource quality driving preferences in consumption, it will also heavily depend on the type of feeding interaction (i.e., the type of consumer search strategy), the ability of the consumer to overcome a prey, and many other aspects. For example, it has been shown in experimental and modeling trials that invertebrate consumers actively switch between larger and smaller prey organisms based on consumer‐resource body size ratios (Kalinkat et al., 2011). Finally, different resources will simply not be available in equal quantities which will result in varying search time for the consumers and additionally constrain relative consumption patterns.

For our purposes, the preference (relative consumption) for consuming different resources can be subdivided into active and passive preferences. Active preference occurs if a consumer assertively chooses to consume more of one and less of other resources. Passive preferences occur when a consumer is driven toward eating more of a given resource due to increased encounter rates, that is, higher relative abundance (availability). In the context of energy‐flux calculations, both concepts can be combined and used at the same time (de Ruiter et al., 1993). Again, in most cases, there will likely be no data on active consumer preference, but they could be available via gut‐content data, observations, or expert knowledge. In fact, many of the methods to obtain information on trophic interactions (see Section 2.4) can help in assigning active preferences and new methods are constantly developed that will help to tackle this issue in the future (see Section 3.3.1). In contrast to active preferences, passive preferences will be easy to assign in the energy‐flux context, because biomass of all nodes is available and thus passive preferences can simply be assigned based on relative resource biomass (Gauzens et al., 2018). Whether it is possible and makes sense to assign such active and passive preferences will depend on the availability of data and the type of interactions in a given community. When there is no information on active preferences, two standard options would be to either (a) assign equal preferences to all resources (a null‐assumption, Barnes et al., 2014) or (b) use passive preferences defined by resource relative abundance or biomass in the given community (Gauzens et al., 2018). One issue that has come up repeatedly over the past years when calculating energy fluxes and using relative resource biomass to define passive preferences is the issue of omnivores feeding on animal and detritus or animal and plant material. In most systems, there will always be a strong overabundance of plants/detritus relative to animal prey, but it seems unlikely that relative consumption follows this relative biomass pattern. In such cases, passive preferences for plant and detritus resources could be manually adjusted to, for example, equal that of the animal resources. It should be noted, however, that such an approach might directly affect the conclusions drawn from a given study and should be adopted with caution if, for example, the goal of a paper is to compare detritivory or herbivory along ecological gradients. In summary, whenever we have information on both active and passive preferences, these can easily be combined (de Ruiter et al., 1993). No matter which option is chosen, it is recommended to discuss the choice in a caveat section. If in doubt, the impact of the preference choice on the conclusions of a given analysis can be tested in a sensitivity analysis (see e.g., Barnes et al., 2020).

In *fluxweb*, preferences can either be calculated outside of the *fluxing* function (taking both active and passive preferences into account if desired) and then supplied to the function, or alternatively calculated by the *fluxing* function itself. If preferences are estimated externally, they have to be supplied to the function by providing nonbinary values in the *mat* argument and setting the *bioms.prefs* argument to *FALSE*. If the choice is to let the *fluxing* function compute the preferences, then active and passive preferences have to be explicitly provided. Active preferences are given by providing non binary values to the *mat* argument. The function then uses the ratio between the different preference values as all of them will be rescaled so that the sum of active preferences is equal to one for each consumer node. Passive preferences, if desired, are automatically calculated by setting the *bioms.prefs* argument to *TRUE*. In this case, the node biomass is used (in combination with active preferences, if provided) following equation (9) in Gauzens et al. (2018). When the choice is to use passive preferences only, the solution is to provide binary values to the *mat* argument while setting the *bioms.prefs* argument to *TRUE*. Please note that, although fluxweb allows cannibalistic links, if they are present in the focal food web, active preferences should be applied to down‐weight cannibalism when also using biomass‐dependent preferences (see Barnes et al., 2020 for an example).

### 2.6 Assimilation efficiency

**FIGURE 6:**
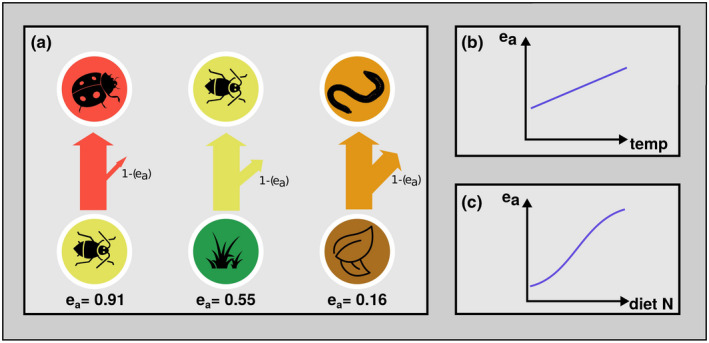
Assimilation efficiency is the proportion of ingested energy that is taken up through the gut walls, that is not egested. In trophic ecology, it is commonly ascribed based on the resource type. Panel (a) shows examples for assimilation efficiency (*e*_a_) for carnivores (eating heterotrophs), herbivores (eating autotrophs), and detritivores (eating detritus) taken from (Lang et al., 2017). Diagonal arrows out of the flux arrow illustrate proportional losses due to assimilation efficiency (1−*e*_a_). (b and c) show two examples of reported drivers of assimilation efficiency, namely, temperature (Lang et al., 2017) and diet nitrogen content (Jochum et al., 2017). For icon sources, please refer to acknowledgements

Traditionally, trophic ecology views ecosystems as hierarchical systems of trophic levels taking up and transforming energy from one level below and transferring it to the next level above (Lindeman, 1942; Odum, 1968). This energetic view heavily relies on energetic efficiencies (Andersen et al., 2009). In short, life on earth is predominantly driven by inputs of solar energy that is taken up by autotrophs using photosynthesis who transform energy into organic materials that is then consumed by primary consumers that are in turn eaten by secondary consumers and so on. Along this food chain (or network), productivity decreases with trophic level, because not all energy produced at the lower level is consumed by the upper level (consumption efficiency), not all energy consumed is assimilated through consumer gut walls (assimilation efficiency) and not all energy assimilated is used to produce biomass (production efficiency). The product of these three efficiencies is the trophic transfer efficiency (Begon, 2006). The efficiencies are typically provided as percentages (0%–100%) or proportions (0–1) (Lang et al., 2017). If we rely on an energy (metabolism)‐centered view, we need to account for the transfer of energy taken from a resource node to a consumer node, including its energetic losses during the conversion. As we go from energy taken out of the resources to energy that is assimilated (used for metabolism and biomass build up) through the gut walls of the consumers, we only need to account for the loss of energy via consumer excretion. This is taken care of by using only assimilation efficiency (Figure 6). If we were to use a biomass‐centered approach (also possible in *fluxweb*), we would need to account for all losses between biomass leaving the resource node and biomass being built up in the consumer node. We would then need to account for both assimilation efficiency and production efficiency. Here, we focus on the metabolism‐centered approach as it accounts not only for the biomass being transferred, but also for the fact that the energy available per unit biomass varies depending on the temperature and organism body mass (metabolic theory of ecology, Brown et al., 2004). Thus, while biomass might be a good indicator of potential energy, it is a poor predictor of what energy it actually provides for the consumer. As we will see, assimilation efficiency varies with the quality of the respective resource for the respective consumer, that is, it depends on consumer and resource identity and the resulting resource quality (suitability) for the consumer (Lang et al., 2017). Additionally, assimilation efficiency varies with temperature (higher temperature—higher assimilation efficiency; Lang et al., 2017) and resource stoichiometry (higher N content—higher assimilation efficiency; Jochum et al., 2017) and likely a whole set of physiological or ontogenetic (consumer life stage and age, physiological adjustment to digest certain resources, etc.), and resource‐structural (plant defenses, indigestible compounds, etc.) aspects.

To calculate energy flux through ecological networks, it is therefore essential to incorporate losses due to assimilation efficiency. We need to assign an assimilation efficiency to every trophic link. This can either be done based on the consumer (all resources consumed by this consumer are taken up with a certain efficiency) or, more commonly, based on the resource (all consumers eating this resource do it with a certain efficiency). It is usually preferred to assign assimilation efficiencies based on the type of resource, as not all consumers feed on only one type of resource, and in these cases a detritus or autotroph resource will not allow the same assimilation efficiency as a heterotroph resource (Lang et al., 2017). Of course, such efficiencies can also be applied based on more specific knowledge, for example, if we happen to know that a certain herbivore consumes a certain plant species with a measured assimilation efficiency. However, this level of precision will only very rarely be available, so here we focus on assigning assimilation efficiencies on a more coarse level. It is possible to assign assimilation efficiencies based on a combination of traits, such as resource type and ambient temperature, and for every single species–species interaction in a fully resolved food web. Most previous work focusing on whole communities seems to take a coarser approach and assign assimilation efficiencies based solely on the resource type. As such, all links including a detritus resource, an autotroph resource, or a heterotroph resource get assigned a literature‐derived average assimilation efficiency (e.g., 0.158, 0.545, and 0.906 at 20°C, respectively (Lang et al., 2017)).

Please refer to Appendix S1: Section 6, for R‐code on assimilation efficiency calculation for arthropod consumers including equations for temperature‐dependent assimilation efficiencies (based on Lang et al., 2017). Table 3 provides examples of literature resources for assimilation‐efficiency estimates of various taxa. In *fluxweb*, assimilation efficiency is applied via a vector or array supplied to argument *efficiencies* in the *fluxing* function as the proportion of consumed energy that is assimilated into the consumer node to then be used for building biomass and respiration, rather than being excreted (Gauzens et al., 2018). Note that such efficiencies can be defined from a resource (the proportion that any consumer would take out of this specific type of resource, default behavior of fluxweb) or consumer perspective (the proportion of energy that a specific consumer would take out of any resource), in *fluxweb* this is specified via argument *ef.level* in the *fluxing* function.

**TABLE 3:** Example literature sources for assimilation efficiencies of different taxa

Taxa	Details/Focus	Reference
Collembola, Acari, Nematoda, Amoebae, Flagellates	Shortgrass prairie, soil fauna	Hunt et al. (1987)
Terrestrial arthropod carnivores, herbivores & detritivores	Temperature dependency	Lang et al. (2017)
Aquatic insects	Resource‐N content dependency	Pandian and Marian (1986)
Fishes	Absorption efficiency, >50 species	Pandian and Marian (1985)
Birds	Food‐type and species dependency	Castro et al. (1989)
Land snails	Woodlands	Mason (1970)

### 2.7 Common issues

#### 2.7.1 Units, logarithms, and regression equations

When calculating energy flux through trophic networks using the food‐web energetics approach, we rely on many different aspects of ecological communities, such as body mass and metabolism. As we have seen above, these variables can often be calculated based on literature‐derived information on regressions, for example, length‐mass regressions or mass‐metabolism regressions. Additionally, when compiling information for whole communities, we often rely on different sources for different taxa. As such, our approach, and the effectiveness of energy‐flux calculations in multitrophic communities, heavily relies not only on the availability of such literature data but also on the efficient written communication of calculation details. One such example for the importance of effective communication between authors and readers of scientific literature is the units of in‐ and output data of regressions. Unfortunately, it is often quite time‐consuming, and sometimes even impossible for the reader, to extract the relevant information on input and output units from papers, for example, on length‐mass regressions. The same is true for dry and fresh masses in ecological papers. Different subdisciplines of ecology and biology seem to be focusing on either dry or fresh body mass which leads to papers lacking the important piece of information telling the reader if the paper uses fresh or dry body mass. As another example, metabolic rates can be expressed in very different units and these often require careful conversions (e.g., of ml O_2_ to J, see Barnes et al., 2018).

Similarly, papers making use of logarithms often do not efficiently report which type of logarithm they use. This issue is additionally complicated by different software using the same function commands for different types of logarithms (natural log and log_10_ in MS Excel and R, for example—in MS Excel, log is decadal logarithm, in R log is natural logarithm). While these issues are quite trivial to solve for the authors of scientific literature, they can turn into an unsolvable issue for their readers, especially if the respective papers are several decades old and the original authors cannot be contacted anymore. Obviously, these issues are not constrained to people calculating energy flux, but given the common dependence of our calculations on other people's data and literature sources and the fact that, for example, physiological papers have often been published decades ago, these issues are of importance here. We therefore recommend to provide very detailed explanations of each individual step taken and the literature resources used for the calculation of energy fluxes. Even if this is not possible to be comprehensively described in the main text of a manuscript, the [Link], [Link] of any energy‐flux paper should ideally provide the level of detail to reproduce every single step.

#### 2.7.2 Getting everything to the same scale

When calculating energy flux, we need to know which organisms are present in our focal system and we need quantitative information on how many of these individuals (densities) interact with each other. This is typically done via sampling a given area or volume and assuming that the sampled organisms constitute the bulk of this interacting community (compare Section 2.1.1). As explained above, there are several issues with this. First, there can be (and often are) organisms feeding in the focal system but not included in our sampling (e.g., because they enter and leave our focal system repeatedly or because the spatial scale they are operating at differs from the scale of our sampling). If we can estimate the fraction of these organisms' energy demand supported by our focal system, we can include these organisms, for example, by setting their individual density to a fraction of 1 per unit area. Alternatively, their impact would have to be ignored in the calculation of energy flux (but could be discussed for our focal system), but it is likely that even a low abundance or short foraging time of a large consumer in our focal system might have a considerable impact on energy flux. Second, several methods of assessing community data are not quantitative in respect to a given standardized area or volume. Such techniques for example include pitfall traps, flight interception traps, but also acoustic or visual assessment of biodiversity in an area. These methods might deliver information on which organisms are present, but without their densities, we cannot directly use them in our assessments of energy flux. That said, we can use such qualitative information to complement available quantitative data, for example, which diversity of a bird community (qualitative data from point counts) our invertebrate networks might support.

## 3 CHALLENGES AND RESEARCH FRONTIERS

Now that we have established how energy flux can be calculated and what it is good for, we would like to highlight a number of challenges that need to be overcome to more effectively use energy flux in ecological research. We present a list of—what we perceive as—promising research frontiers for future energy‐flux research.

### 3.1 Flux as a relative quantity versus actual numerical correctness

First of all, it is important to reiterate that calculated energy fluxes should be perceived as comparative values rather than taken as being numerically correct representations of the energy flowing through a system. This is true for a number of reasons. As we have seen above, the absolute value of calculated flux through an ecological community heavily depends on the somewhat arbitrary decision of what we define as the top predator or highest trophic level (which is also linked to the spatial scale of the assessment). Because the energy needed at such a high‐trophic level needs to be channeled through all levels below, including assimilation losses at each conversion, the additional consideration of even a very small top‐predator population can heavily affect the absolute value of calculated energy flux (hence the importance of higher‐level consumers which are not sampled feeding on our target community). Another reason that has also already been mentioned above is the (typical) use of basal metabolic rates. We know that field metabolic rates are usually a multiple of those base‐rates and thus flux calculations based on basal metabolic rates can be compared to each other but will not necessarily be a good predictor of the true energy flux based on the activity‐cycles of the focal organisms. This does not mean that flux calculations are problematic per se, it simply highlights their use as a comparative quantity, e.g., to be compared among treatments in standardized experiments, or well‐defined subsets of the trophic ladder in different systems.

### 3.2 Testing the sensitivity of the method

While flux calculations based on the food‐web energetics approach have been related to observed fluxes before (Neutel & Thorne, 2014), this step towards further validating the approach will not be simple. The reason for this is that when comparing, for example, traditional measures of herbivory to calculated fluxes to herbivores, both approaches have their weaknesses and it is not simple to decide which measure should be the benchmark to compare the other against. However, at the very least it would be interesting to know if and how strongly different measures of ecosystem processes correlate with estimations of energy flux, when and where they do not, and why.

As mentioned repeatedly throughout the above sections, we do not know much about the sensitivity of the energy‐flux calculations to several methodological decisions along the way. Examples are the impact of using average body mass versus more‐detailed body size distributions, or the aggregation of food‐web topology. These aspects need to be further investigated to provide advice for future research on energy flux. Assessing the potential impact of topological aggregation and the resolution of other parameters, such as assimilation efficiency or metabolic rates (see *fluxweb* function *sensitivity* in this regard), can be done using analytical simulations based on simulated food‐web and community data. However, it will be just as interesting to test the effect of real‐world variation in various parameters such as the body‐size distributions of different trophic groups on the resulting energy flux.

### 3.3 Way forward—promising frontiers

Previous work has discussed how energy flux can be used in future biodiversity‐ecosystem functioning research (Barnes et al., 2018). Here, we thus focus on methodological advances in and the broad applicability of calculating energy flux.

#### 3.3.1 Topology and active preferences: better assessment and embracing variability

Both food‐web topology and active consumer preferences (and not only the presence of taxa or their biomass—availability as a resource) change with various external drivers, such as temperature, resource quality, changing predator–prey body size ratios (Ushio et al., 2018). As such, both these features will regularly change across experimental treatments or environmental gradients inherent in observational studies. However, when comparing energy flux through trophic networks, such changes are often ignored because of a sheer lack of available information (we hardly know who feeds on whom—knowing how this changes due to abiotic or biotic conditions is usually out of reach). As such, the topology and active preferences we use to compare energy flux through communities, for example, in forest litter of different tropical land‐use systems (Barnes et al., 2014) or in grassland BEF experiments on different continents (Barnes et al., 2020), are usually fixed and preferences are affected only through changes in passive preferences, that is, local relative availability of different resources. However, this issue can be overcome by more‐widely adopting high‐throughput techniques of assessing trophic relationships (Brose & Scheu, 2014). Using molecular gut content analysis (Eitzinger et al., 2018), metabarcoding (Casey et al., 2019; Oliverio et al., 2018; Sousa et al., 2019) fatty‐acid analysis (Ferlian & Scheu, 2014; Ferlian et al., 2012; Ruess et al., 2005), compound‐specific and bulk stable‐isotope analysis (Lesser et al., 2020; Potapov et al., 2019b), or combinations of different techniques (Potapov et al., 2020) will help to unravel such changes in topology and consumer preferences.

#### 3.3.2 Integrating functional traits

Functional traits are increasingly being used in ecology. While plant functional traits have been widely used and centrally available over the past decades (Díaz et al., 2015; Kattge et al., 2011), their use is becoming more readily available across different branches of the Tree of Life (Gallagher et al., 2020) even though centralized platforms comparable to the plant realm are still lacking for animals (Schneider et al., 2019). Functional traits have been found to structure trophic networks (Laigle et al., 2017) and are good predictors of feeding interactions (Brose et al., 2019; Brousseau et al., 2018). Consumer and resource traits can be used to assign feeding links and weight feeding preferences. They affect organism energy demand and assimilation efficiency. While the theoretical connection of functional traits to these central aspects of energy‐flux calculations is well established, it seems that the full potential of actually using trait data to inform energy‐flux calculations is not comprehensively taken advantage of, yet. Examples include the use of consumer‐resource body size ratios for defining topology and preferences (Brose et al., 2019; Hines et al., 2019), and stoichiometric mismatch for informing feeding preferences, assimilation efficiency (Jochum et al., 2017), and even consumer metabolism (Jeyasingh, 2007).

#### 3.3.3 Realistic metabolic rates: basal vs. field metabolic rates; taking behavior and its climate dependence into account

We have seen that metabolic rates are very important in the adapted food‐web energetics approach. Field metabolic rates are usually by a factor of three higher than basal rates (Savage, Gillooly, Woodruff, et al., 2004). It would be interesting to assess how using field metabolic rates, rather than basal rates, affects the outcome of flux calculations. Because of the cascading nature of how changes at different trophic levels affect flux calculations, using field metabolic rates would not just compare to using in a simple factor of how total energy flux is affected, but its effect on the resulting energy‐flux estimation will depend on the given food web topology and body size structure. However, it seems that field metabolic rates are simply not as easily available as basal rates (but see Hudson et al., 2013).

We know that metabolic demand is highly sensitive to temperature, with higher temperatures leading to higher metabolic demands. However, this is not the only way in which changing climate and other abiotic and biotic drivers will affect organism energy demands, or energy fluxes more generally. Specifically, changes in animal behavior, due to different abiotic or biotic environmental conditions, are likely to alter network topology, feeding preferences, and metabolic rates (Barton & Schmitz, 2009; Hawlena & Schmitz, 2010). As such, changing conditions will not only physiologically affect respiration but also alter organism behavior, such as movement patterns with consequences for field metabolic rates, or time spent on predator‐avoiding behavior (Schmitz, 2008), which might affect food‐web topology and preferences.

#### 3.3.4 Exploring elemental fluxes

As mentioned above, there are multiple ways in which the concept of ecological stoichiometry (Sterner & Elser, 2002) can be applied to facilitate energy‐flux calculations (Barnes et al., 2018). In addition to using stoichiometry to inform estimations of metabolic rate, feeding preferences, or assimilation efficiencies, the whole concept of calculating energy and matter fluxes through trophic networks can be applied to assess elemental fluxes instead of, or in addition to, energetic fluxes (Barnes et al., 2018). Recent advances in this direction have for example been made for fish (Schiettekatte et al., 2020), but there is plenty of scope to make these options available across taxa to enable a whole new suite of exciting questions to be answered at the interface of food‐web ecology, ecological stoichiometry, and the study of energetics in community and ecosystem ecology.

## CONFLICT OF INTEREST

The authors declare no conflict of interest.

## AUTHOR CONTRIBUTIONS

**Malte Jochum:** Conceptualization (lead); Investigation (lead); Methodology (lead); Software (lead); Visualization (lead); Writing‐original draft (lead); Writing‐review & editing (lead). **Andrew D. Barnes:** Conceptualization (equal); Investigation (equal); Methodology (equal); Visualization (supporting); Writing‐review & editing (equal). **Ulrich Brose:** Conceptualization (equal); Investigation (equal); Methodology (equal); Writing‐review & editing (equal). **Benoit Gauzens:** Conceptualization (equal); Investigation (equal); Methodology (equal); Software (equal); Visualization (supporting); Writing‐review & editing (equal). **Marie Sünnemann:** Investigation (equal); Writing‐review & editing (equal). **Angelos Amyntas:** Investigation (equal); Writing‐review & editing (equal). **Nico Eisenhauer:** Funding acquisition (lead); Investigation (equal); Methodology (equal); Writing‐review & editing (equal).

## Supporting information

Appendix S1

Appendix S2

## Data Availability

Associated R‐code is available as Appendices S1 and S2 to this article. There are no data associated with this article.
