# Supplementary material for: For flux's sake: General considerations for energy‐flux calculations in ecological communities
Source: Ecol Evol. 2021 Sep 14;11(19):12948–69. doi: 10.1002/ece3.8060 (PMC8495806; doi:10.1002/ece3.8060)
Supplement: Supplementary file 1 — Appendix S1 [file ECE3-11-12948-s001.pdf]

Appendix R-Code for ‘For flux’s sake: General considerations for energy-flux calculations in ecological communities’

An introduction for those new to the method or struggling with its implementation

Malte Jochum Andrew D. Barnes Ulrich Brose Benoit Gauzens
Marie Sünnemann Angelos Amyntas Nico Eisenhauer

Corresponding author: Malte Jochum (malte.jochum@idiv.de)

Introduction

Purpose of this script

The purpose of this script is to facilitate the calculation of parameters required or useful for the calculation of energy fluxes through trophic networks. It is based on available literature resources and simply provides R-code examples of how to implement the calculation of metabolic rates based on animal body mass and temperature (Ehnes, Rall & Brose (2011), Brown *et al.* (2004)), the calculation of invertebrate body mass based on body length (Sohlström *et al.* (2018), Sohlström & Jochum (2021), Mercer *et al.* (2001)), and the calculation of temperature-specific assimilation efficiencies (Lang *et al.* (2017)).

This script has been streamlined and annotated to the best of my knowledge. Please direct questions and reports of bugs or missing information to me via malte.jochum(at)idiv.de.

Last time performed:

01.09.2021, by Malte Jochum

Software

R version 3.6.3 (2020-02-29) – "Holding the Windsock" Copyright (C) 2020 The R Foundation for Statistical Computing Platform: x86_64-pc-linux-gnu (64-bit)

Practical considerations

Please note that section numbering has been set up to match the respective sections in the main text.

2. Energy loss

2.1 Metabolic demand

In the adapted food web energetics approach, energy loss from network nodes is quantified via organism metabolic demand.

Calculating arthropod metabolic rates from body mass and temperature

Metabolic rates for terrestrial invertebrate macrofauna and mesofauna can, for example, be calculated using taxon-specific regressions from Ehnes *et al.* (2011). These regressions calculate per capita metabolic rates based on organism body mass and temperature. Note that Ehnes *et al.* (2011) group Collembola with other insects, but provide specific metabolic rate regressions for Oribatida, Prostigmata, and Mesostigmata.

```
rm(list=ls())

#Form of Ehnes Equation: lnI = lni0 + a*log(M) - E*(1/(kT))

# Regression parameters from Ehnes 2011 (Ecol Letters), Table 2, Phylogenetic model,
# and additional regression parameters from Roswitha Ehnes (unpublished, pers. communication).
# Order of regression parameters: lni0, a, E
Arapar <- c(24.581475, 0.5652537, 0.7093476) # Regression parameters for Arachnida
Colpar <- c(21.41811, 0.73755, 0.63938) # Parameters for Coleoptera, personal communication
Inspar <- c(21.97205, 0.7588950, 0.6574038) # Parameters for Insecta (see Ehnes for what is included)
```

```r
###
#Set temperature, in Kelvin -> add 273.15 to your temperature values in °C:
tempC <- 20 # temperature in °C

tempK <- 273.15+tempC

# set example body mass (fresh mass, in mg)
# Make sure to (ideally) stay within the body mass range of regressions. Here, refer to Ehnes 2011 Table 1.

ara_bodymasses <- c(1.2, 25.8, 500) #Araneae

col_bodymasses <- c(25.9, 80.7, 600) #Coleoptera

ins_bodymasses <- c(0.05, 17.3, 100, 6000) #Insect

k = 8.62e-05 # Boltzmann's constant

# Calculate per-capita metabolic rates in W (see unit conversion below) for example body masses of spiders,
# beetles, and insects (in general)
ara_metabolicrates <- exp(Arapar[1]+Arapar[2]*log(ara_bodymasses)-Arapar[3]*(1/(k*tempK)))/3600

col_metabolicrates <- exp(Colpar[1]+Colpar[2]*log(col_bodymasses)-Colpar[3]*(1/(k*tempK)))/3600

ins_metabolicrates <- exp(Inspar[1]+Inspar[2]*log(ins_bodymasses)-Inspar[3]*(1/(k*tempK)))/3600

# Unit conversions:
#logI = lni0+a*log(bodymass)-E*(1/(k*temp)) # Ehnes formula
#I <- exp(lni0+a*log(bodymass)-E*(1/(k*temp))) # metabolic rate in J/h
#IW <- exp(lni0+a*log(bodymass)-E*(1/(k*temp)))/3600 # metabolic rate in W

#IW <- exp(Arapar[1]+Arapar[2]*log(bodymass)-Arapar[3]*(1/(k*temp)))/3600 # example calculation
```

Alternatively, use metabolic-theory scaling based on Brown *et al.* (2004). Here, we use the parameters for invertebrates; for other parameters, see their Figure 1B.

```r
# Calculate per-capita metabolic rates in W for body masses in g for invertebrates.
# Temperature needs to be supplied in °C, the code below converts this to Kelvin.

rm(list=ls())

boltz <- 0.00008617343 # Boltzmann's constant

temp <- 20 # temperature in °C

metabolicrates <- exp((0.71 * log(0.1) + 17.17) - (0.69/(boltz*(273.15+temp)))) # example mass: 0.1 g (100 mg)

# Note that, in this format, the equation also results in per-capita metabolic rates. If you are ever unsure
# of this, plot metabolic rates versus body masses: For per-capita metabolic rates, this should be a
# positive, saturating relationship. If you get a negative relationship, you probably have an equation
# providing per-g-biomass metabolic rates.
```

As we can see from the above examples, using regressions from two different sources and based on different datasets can result in quite different metabolic rates. It is therefore important to ideally not combine regressions from different sources in one project but instead try and either use general metabolic scaling for all organisms or use more specific regressions for the respective taxa in a given study. However, if combining various taxa, this might require different metabolic-rate sources which again leads to potential issues in comparability of resulting metabolic demand estimations.

3. Body mass

3.1 Assessing body mass

Calculating arthropod fresh body mass from body length

Many papers offer regression parameters for calculating body mass based on various morphological measurements. Here, we provide a recent example for calculating terrestrial arthropod body masses. Sohlström *et al.* (2018) provide regressions for several arthropod macrofauna taxa, for just body length or both body length and width, and for both temperate and tropical arthropods. Calculating fresh body mass based on length (mm) and taxonomic group, for temperate spiders and staphylinid beetles, works as shown below.

Note that the regression coefficient table that we load below is based on the main text and supporting information of Sohlström *et al.* (2018) and has recently been published in the iDiv Data Repository (Sohlström & Jochum (2021) https://doi.org/10.25829/idiv.3483-9-2307). Raw data for Sohlström *et al.* (2018) is available via Dryad (https://doi.org/10.5061/dryad.vk24fr1). Consequently, it is also possible to derive regression parameters for taxa that the original paper did not further analyze. Alternatively, body length mass data could be complemented with data from other sources for improved sample size or extended body-length range covered by a resulting regression.

```
rm(list=ls())

# Set to your local folder
setwd("...")
#setwd("/home/malte/projects/2020_General_FLUX_considerations/markdown/code")

# Load Sohlstroem 2018 regression parameters (extracted from main text and SI tables)
regdat <- read.csv("./3483_9_Sohlstroem_Full_regression_parameters.csv", sep = "," )

unique(regdat$taxon) # check for which taxa Sohlström et al. provide regressions

# In the following example code, we use example individual-level body lengths in mm to calculate
# per capita, fresh body mass in mg. The example assumes temperate animals and uses model type LTR,
# so information on length, taxon and geographic region (see Sohlstroem et al. 2018).

# Check Table 2 in Sohlstroem et al. 2018, and for taxa not analzed in the main text also the data on Dryad,
# for body-length ranges of the desired taxa to make sure that your animals fall within that range (ideally).

# Araneae (order-level regression for spiders)
araneae_body_lengths <- c(5.4, 8.5, 11.8) # example body lengths

araneae_fresh_bodymasses <- 10^(regdat$intercept[regdat$subset=="main"
  & regdat$model=="LTR" & regdat$taxon=="Araneae" & regdat$region=="temperate"]
    + regdat$slope_l[regdat$subset=="main" & regdat$model=="LTR"
    & regdat$taxon=="Araneae" & regdat$region=="temperate"]
    * log10(araneae_body_lengths))

# Staphylinidae (family-level regression for staphylinid beetles)
staphylinidae_body_lengths <- c(2.1, 8.5, 23.8) # example body lengths

staphylinidae_fresh_bodymasses <- 10^(regdat$intercept[regdat$subset=="appendix"
  & regdat$model=="LTR" & regdat$taxon=="Coleoptera (Staphylinidae)" & regdat$region=="temperate"]
    + regdat$slope_l[regdat$subset=="appendix" & regdat$model=="LTR"
    & regdat$taxon=="Coleoptera (Staphylinidae)" & regdat$region=="temperate"]
    * log10(staphylinidae_body_lengths))
```

Soil-mesofauna body masses can, for example, be calculated using regressions from Mercer *et al.* (2001).

```
#Mercer regressions provide fresh body mass in µg from length in mm, thus we transform the calculated values
# to mg by dividing by 1000. Careful: this differs for different regressions in Mercer!

# In the following example code, we use example individual-level body lengths in mm to calculate
```

```r
# per capita, fresh body mass in mg (hence the factor, see above).

# Check the tables in Mercer et al. 2001 for body-length ranges of the desired taxa to make sure that your
# animals fall within that range (ideally).

rm(list=ls())

# Prostigmata (L-FM (Mercer; Prostigmata regression))
prostigmata_body_lengths <- c(0.3, 0.5, 1.0) # example body lengths

prostigmata_fresh_bodymasses <- (10^(2.124 + 2.808 * log10(prostigmata_body_lengths)))/1000
# divided by 1000 as this Mercer regression provides µg, but we want mg

# Collembola (L-FM (Mercer; Collembola ALL regression))
collembola_body_lengths <- c(0.7, 1.5, 3.2) # example body lengths

collembola_fresh_bodymasses <- (10^(1.339 + 1.992 * log10(collembola_body_lengths)))/1000
# divided by 1000 as this mercer regression gives µg, but I want mg

#In Mercer et al. 2001, there are different mass units used for diferent taxa in the same table (!),
# AND they do not openly say if their "log" is log10 or ln. This information be taken from the text though
# as they say others have used ln instead and mark this as a difference from their approach.
```

6. Assimilation efficiency

Here, we calculate assimilation efficiencies for different types of resources (animal, plant, detritus) and in dependence of temperature based on Lang *et al.* (2017). Ideally, check Figure 2a in Lang *et al.* (2017) to make sure you do not predict assimilation efficiencies for temperature-consumer combinations they do not cover in their models, e.g. for the combination of detritivores and 30°C and higher.

```r
rm(list=ls())

# Input parameters
T0 <- 273.15 + 20
boltz <- 0.00008617343 # Boltzmann's constant

temp <- 22 # Here, you need to adjust to your desired target temperature (in °C).

temp.kT <- ((273.15+temp)-T0)/(boltz*(273.15+temp)*T0)

# Calculate temperature-corrected assimilation efficiencies, based on parameters taken
# from Lang et al. 2017 (Oikos), Appendix Table A9.
eass_animal <- exp(2.266)*exp(0.164*temp.kT) / (1 + exp(2.266)*exp(0.164*temp.kT))

eass_plant <- exp(0.179)*exp(0.164*temp.kT) / (1 + exp(0.179)*exp(0.164*temp.kT))

eass_detritus <- exp(-1.670)*exp(0.164*temp.kT) / (1 + exp(-1.670)*exp(0.164*temp.kT))
```

The values provided in the Lang *et al.* (2017) main text ("Carnivores had the highest assimilation efficiency 0.906, herbivores had an intermediate assimilation efficiency 0.545, and detritivores had the lowest assimilation efficiency 0.158, note that we report the inverse logit here") are for a temperature of 20°C.

Literature cited

Brown, J.H., Gillooly, J.F., Allen, A.P., Savage, V.M. & West, G.B. (2004) Toward a metabolic theory of ecology. *Ecology*, **85**, 1771–1789.

Ehnes, R.B., Rall, B.C. & Brose, U. (2011) Phylogenetic grouping, curvature and metabolic scaling in terrestrial invertebrates. *Ecology Letters*, **14**, 993–1000.

Lang, B., Ehnes, R.B., Brose, U. & Rall, B.C. (2017) Temperature and consumer type dependencies of energy flows in natural communities - a database analysis. *Oikos*, **4**, 1–9.

Mercer, R.D., Gabriel, A., Barendse, J., Marshall, D. & Chown, S. (2001) Invertebrate body sizes from Marion Island. *Antarctic Science*, **13**, 135–143.

Sohlström, E. & Jochum, M. (2021) Arthropod allometric regression parameters from Sohlström et al. 2018.

Sohlström, E.H., Marian, L., Barnes, A.D., Haneda, N.F., Scheu, S., Rall, B.C., Brose, U. & Jochum, M. (2018) Applying generalized allometric regressions to predict live body mass of tropical and temperate arthropods. *Ecology and Evolution*, **8**, 12737–12749.
